# Online Captive: The Impact of Social Media Addiction on Depression and Anxiety—An SEM Approach to the Mediating Role of Self-Esteem and the Moderating Effects of Age and Professional Status

**DOI:** 10.3390/bs15040481

**Published:** 2025-04-07

**Authors:** Daniela-Elena Lițan

**Affiliations:** Psychology Department, West University of Timișoara, 300223 Timișoara, Romania; daniela.litan@e-uvt.ro

**Keywords:** social networks, depression, anxiety, self-esteem, professional status

## Abstract

In the “era” in which social networks have become an integral part of everyday life, this study aims to analyze the impact of social network addiction on mental health, with a focus on the manifestation of anxiety and depression symptoms. The relationship analyzed on a batch of a Romanian adult population, explained by self-esteem and influenced by factors such as age and professional status, highlights the fact that all age categories and professional status can be affected by addiction to the online environment but with different degrees of intensity. The analyses carried out using Structural Equation Modeling (SEM) suggest that young people and professionally inactive people are the categories with the highest degree of self-esteem impairment and with a high potential to manifest symptoms associated with depression and anxiety disorders, as a result of the intense use of social networks. The current study makes a significant contribution to the specialized literature, given the small volume of similar studies conducted on the adult population of Romania.

## 1. Introduction

With a global popularity of 66.76% in 2024 (Statista, 2023), social networks have become an important part of our lives, always at our fingertips to interact with people we know, make new friends, express ourselves or stay up to date with the latest news (Ursoniu et al., 2022). Using social networks is one of the most popular activities online, with over 5.17 billion users in 2024 and expected to reach 6.05 billion users in 2028 (Statista, 2024). Developed and used both as websites and as mobile phone applications, social media platforms, such as YouTube, Facebook, Twitter, Instagram, WhatsApp, Telegram and many others (Jena et al., 2024), are among the most popular online platforms in recent years.

However, beyond the “glamor” portrayed by the social networks, their intense and increasing use (Zewude et al., 2025) can also hide numerous risks or potential damage to mental health, from symptoms of anxiety and depression, the pressure of social comparison with others, poor sleep patterns, social isolation, to the risk of suicide (Naslund et al., 2020; Beyari, 2023; Zsila & Reyes, 2023) or even misinformation (Suarez-Lledo & Alvarez-Galvez, 2021). Under the conditions of the intense use of social networks, a series of cognitive and behavioral mechanisms are activated and strengthened, influencing self-perception, interpersonal relationships and emotional well-being, generating chain consequences such as those mentioned above. The cognitive and behavioral mechanisms triggered in this context can be explained, starting primarily from the social comparison theory (Festinger, 1954), which describes the causes and consequences of comparison with others (Neighbors et al., 2013). According to this theory, social comparison refers to the processes by which individuals evaluate their own abilities, opinions, attitudes, feelings, physical traits, achievements, or any other aspects of themselves in relation to other individuals and/or groups (Guyer & Vaughan-Johnston, 2018), and, in the “digital age”, social networks provide an ideal environment for this type of comparison, even amplifying its consequences. Over time, the social comparison theory has acquired new valences, for example, we find upward social comparison, that is, comparison with more successful people, and downward social comparison, that is, comparison with weaker people, in the specialized literature (Wang et al., 2017; Guyer & Vaughan-Johnston, 2018). Of course, currently, we also find the social comparison theory associated with the digital environment and social networks in the literature (Vogel et al., 2014; Qiu, 2024; Verduyn et al., 2020), represented by the following examples, without limiting the literature to these:

- Direct and upward social comparison through constant exposure to unrealistic standards of success, beauty, edited images and idealized lifestyles (Calogero et al., 2005; Tian et al., 2024; Merino et al., 2024);
- Downward comparison and the need for validation that can, for example, generate dependence on likes and comments to validate personal value (Ballara, 2023; Lee et al., 2020);
- FOMO (Fear of Missing Out) and upward comparison: People addicted to social networks feel anxiety and stress when they are not online, fearing that they are missing out on important events, thus generating feelings of inadequacy and exclusion (Alabri, 2022);
- The polarization of opinions and emotional radicalization: Social media algorithms create an environment in which users are exposed to content that confirms their beliefs (this also includes the online groups to which a person belongs and with which they compare themselves) (Pranesh & Gupta, 2024; Akram & Nasar, 2023; Cinelli et al., 2020), which can amplify negative emotions;
- Decreased concentration and information overload: Constant exposure to online stimuli creates an environment of rapid and fragmented comparison. Beyond this aspect, information overload reduces self-efficacy and increases anxiety (K. Li et al., 2024; Qin et al., 2024);
- Emotional desensitization and reduced empathy (Shaista et al., 2024): If users frequently compare themselves to others and feel inferior, they may develop emotional detachment. Beyond this aspect, repeated exposure to various stimuli (positive or negative) in relation to social networks influences users’ perceptions and behavior (Kušen & Strembeck, 2023).

Therefore, as we have shown above, according to the specialized literature, addiction to social networks reduces the ability to regulate emotions, thus generating a favorable ground for the manifestation of symptoms of depression and anxiety. Moreover, self-esteem, a subjective assessment of an individual’s self-worth (Rüther et al., 2023), as a mediator of the relationship (addiction to social networks → anxiety/depression), can diminish or amplify its strength. Low self-esteem scores have been associated with a more intense use of social networks (Hussain et al., 2020; Al Ahbabi et al., 2024) but also with symptoms of depression (Rosenthal & Tobin, 2023) and anxiety (Colak et al., 2023), according to the specialized literature.

However, the effect of the previously described relationship (social media addiction → anxiety/depression mediated by self-esteem) is moderated by age and professional status. This statement is supported by the self-determination theory, which assumes that people are inherently inclined towards psychological growth and integration (Ryan & Deci, 2020). Therefore, according to the self-determination theory, the satisfaction of fundamental psychological needs—competence, autonomy and social relationships—influences the individual’s resilience faced to the impact of social media. In other words, the level of autonomy and competence, elements that vary according to age (Sheldon et al., 2006) and professional status (Martela & Riekki, 2018), influences the effects of social media use on mental health.

Considering the elements presented above, as well as the fact that the specialized literature is limited in the number of research studies dedicated especially to the region of South-Eastern Europe—Romania, regarding the subject debated in this paper, focusing on the adult population of Romanian citizenship, in this research, we aimed to investigate the relationship between social media addiction and symptoms of depression and anxiety, analyzing self-esteem as a mediating factor, and age and professional status as moderating factors of this relationship. In this context, we propose the following research hypotheses:

**H1:**  *Self-esteem mediates the positive relationship between social media addiction and the manifestation of anxiety disorder symptoms.*

**H2:**  *Age and professional status moderate the mediation effect of self-esteem between social media addiction and the manifestation of anxiety disorder symptoms.*

**H3:**  *Self-esteem mediates the positive relationship between social media addiction and the manifestation of depressive disorder symptoms.*

**H4:**  *Age and professional status moderate the mediation effect of self-esteem between social media addiction and the manifestation of depressive disorder symptoms.*

**Figure 1:**
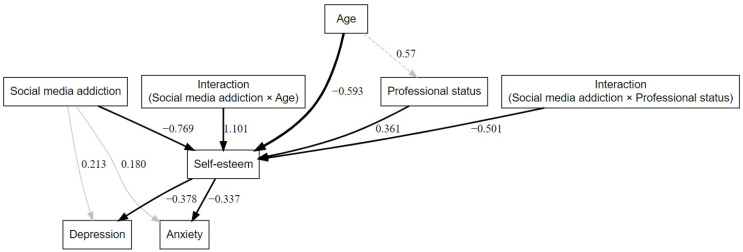
The hypothesized model.

Figure 1 presents the proposed research model.

## 2. Materials and Methods

### 2.1. Study Design

The current study was approved by the Ethics Committee (The Scientific Council of University Research and Creation) from the West University of Timișoara in August 2024, Romania, and was conducted in accordance with the World Medical Association Helsinki declaration. The current study has a cross-sectional survey design, and data were collected between 11 December 2024 and 8 February 2025. All participants in this study were informed about the context, objective and purpose of this study, and all provided informed consent.

### 2.2. Participants

In total, 217 people participated in the current study, out of whom 37.8% were men and 62.2% were women. The participants were adult Romanian citizens, aged between 18 and 62 years (M = 36.15, SD = 11.92), whose professional status varied as follows: 65.9% employed, 1.4% unemployed, 18.4% students, 0.9% housewives, 12.4% self-employed and 0.9% retired.

The last school completed by the study participants ranged from 24% high school, 35.9% college, 35% Master’s degree, to 5.1% doctoral studies. Additionally, all participants were enrolled in a form of formal or non-formal education (e.g., university, training courses, etc.) at the time of completing the questionnaire or shortly before that moment.

The participants live predominantly in urban areas, that is, 80.1%, and 55.1% of them do not have children, while 70.5% of the respondents are in a couple relationship.

The batch size was calculated a priori with the G*Power program, which, for an average effect, a power of 0.80, a type I error equal to 0.05 and 1 predictor displayed a minimum size of 55 participants.

### 2.3. Measures

The survey consisted of several measurement instruments, as follows:

(a) The Romanian version of the Social Media Addiction Scale

The social media addiction was measured using the Romanian version, validated on the Romanian population, of the Social Media Addiction Scale—Student Form (Ursoniu et al., 2022). The scale has 29 questions and assesses the number of hours spent on virtual social media platforms, how this behavior affects mood, whether it causes conflicts in the family or with friends and possible relapses (Ursoniu et al., 2022).

The Social Media Addiction Scale allows participants to record responses on a 5-point Likert scale (from 1—Strongly disagree to 5—Strongly agree).

In the current research, the Social Media Addiction Scale had very good internal consistency, with Cronbach’s alpha value: 0.934.

(b) The DASS-21R Questionnaire (Anxiety, Depression and Stress Assessment Scales)

Anxiety and depression levels were measured using the Anxiety and Depression Scales from the DASS-21R questionnaire (Lovibond & Lovibond, 1995), which was adapted and standardized for the Romanian population (Perţe & Albu, 2011). The questionnaire includes 21 items, distributed on three scales, as follows: anxiety, depression and stress, allowing participants to record responses on a 4-point Likert scale (from 0—did not apply to me, to 3—applied to me very much or almost all the time).

The DASS-21R questionnaire assesses emotional state in relation to situations outside the testing context (not just at the time of testing), identifying the essential features of each syndrome and eliminating the overlap of items between scales, and can be used both in research and in a clinical context (Perţe & Albu, 2011).

The Anxiety and Depression scales of the DASS-21R questionnaire, used in the current research, had good internal consistency, with Cronbach’s alpha values: 0.867 (Anxiety) and 0.869 (Depression).

(c) Rosenberg self-esteem scale

Self-esteem was measured using the Rosenberg Scale (Rosenberg, 1965)—Romanian version. The Rosenberg self-esteem scale consists of 10 items and was adapted to the Romanian population by (Robu et al., 2015). The scale allows participants to record responses on a 4-point Likert scale (from 1—Strongly Agree to 4—Strongly Disagree).

In the current research, the Rosenberg self-esteem scale had very good internal consistency, with a Cronbach’s alpha value of 0.908.

(d) Professional status and age

The participants answered the question: What is your professional status? They had the opportunity to choose one of the following options: student, employee, self-employed, unemployed, housewife and retired (pensioner).

After collecting all the research responses, in order to perform the statistical analysis, their equivalence was achieved, as follows:

- The answer: Student (pupil) was equivalent to the value 1;
- The answer: Employee was equivalent to the value 2;
- The answer: Self-employed was equivalent to the value 3;
- The answer: Unemployed was equivalent to the value 4;
- The answer: Housewife was equivalent to the value 5;
- The answer: Retired (pensioner) was equivalent to the value 6.

In the current study, the participants’ age was calculated based on their response to the requirement to fill in the year (date) of birth.

### 2.4. Procedure

The participants’ responses were collected online, through the Google Forms platform, between 11 December 2024 and 8 February 2025, and the questions were asked in Romanian. The questionnaire was promoted on professional platforms, on social platforms, on mobile messaging platforms and by email to the target groups and was completed by the participants voluntarily and anonymously. The participants gave their informed consent to participate in this study and were informed about the objectives of this research and also that they could withdraw at any time during this study.

The current study was pre-registered on the Open Science Framework platform (objectives, main hypotheses, study design, data collection procedure, measured variables and statistical analysis plan) at https://osf.io/yefdk/?view_only=728f65c5ffbd43ab9818aaaff35abc35, accessed on 9 December 2024, prior to data collection. It is also necessary to mention that the current study is a sub-study of the research topic: digital transformation as a factor in the manifestation of clinical symptoms of depression and anxiety, mediated by the level of self-esteem.

The general questionnaire was composed of demographic questions (year of birth, gender, level of education, professional status, marital status, etc.) followed by the questionnaires (in this order): DASS-21R scales for assessing anxiety and for assessing depression, the Rosenberg Scale for establishing self-esteem and the Romanian version of the Social Media Addiction Scale.

### 2.5. Statistical Analysis

In order to evaluate the proposed model, we performed Structural Equation Modeling (SEM) using the RStudio software product—version 2024.12.0 (build 467)—Windows 10 Pro operating system. The packages used in performing the statistical analysis and graphical representation of the model were lavaan and DiagrammeR (see Figure 1).

SEM is a multivariate data analysis method used to examine complex relationships (Hair et al., 2021) between observed and latent variables while taking into account the measurement error. It is similar but much more powerful than regression analyses (Beran & Violato, 2010).

With a view to evaluating the model proposed in this research (data-model fit), the criteria established in the paper (Hu & Bentler, 1999) were used, namely, the following: Comparative Fit Index (CFI) ≥ 0.96, Standardized Root Mean Square Residual (SRMR) ≤ 1.0, or Root Mean Square Error of Approximation (RMSEA) ≤ 0.06 and Standardized Root Mean Square Residual (SRMR) ≤ 0.08.

## 3. Results

### 3.1. Preliminary Analysis

Table 1 presents the descriptive statistics and correlations between the study variables. By analyzing Table 1, we can see that social media addiction positively and significantly correlates with both anxiety (r = 0.281, *p* < 0.001) and depression (r = 0.327, *p* < 0.001), supporting the central idea of the current study: the excessive use of social media is associated with higher levels of symptoms of anxiety and depression disorders. In support of hypotheses H1 and H3, we find, in Table 1, the correlations: social media addiction—self-esteem: r = 0.302, *p* < 0.001, self-esteem—anxiety: r = −0.391, *p* < 0.001, self-esteem—depression: r = −0.443, *p* < 0.001, suggesting that, although active social media users tend to have higher self-esteem, the effect is not beneficial for everyone, given the negative correlations between self-esteem and anxiety/depression. The previous studies suggest that social media use may have mixed effects on self-esteem: some people benefit from this interaction, even temporarily (Rüther et al., 2023), while others are negatively affected (especially through social comparison and external validation) (M. Jan et al., 2017). Therefore, the aforementioned correlations highlight a potential mediation effect, namely, the excessive use of social media can lead to decreased self-esteem, which, in its turn, can increase anxiety/depression.

In order to make moderation methodologically valid, the correlation between the moderator and the predictor must be weak or insignificant. In support of this methodological criterion as well as hypotheses H2 and H4, we can notice in Table 1 that the correlations between the moderators (professional status, age) and the predictor (social media addiction) are insignificant (professional status—social media addiction) or weak (age—social media addiction).

### 3.2. The Relationship Between Social Media Addiction and the Manifestation of Symptoms of Anxiety and Depression Disorders, and Moderated Mediation

According to the criteria (Hu & Bentler, 1999), the test indicated a good fit of the model, and the theoretical specification of the relationships between the variables is adequate: CFI = 0.991, SRMR = 0.021, RMSEA = 0.053 and 90% CI (0.000, 0.104). Also, CMINF/DF = 1.6, NFI = 0.978, *p*-value (chi-square) = 0.118, df = 18 and TLI = 0.981.

As far as the direct effects are concerned, in relation to the hypotheses of this study, the following results were obtained: addiction to social networks predicts the appearance of symptoms of anxiety disorders (β = 0.180, *p* = 0.004) and depression (β = 0.213, *p* = 0.002). Also, the intense use of social networks has a significant negative effect on self-esteem (β = −0.769, *p* = 0.001). This situation, the decrease in self-esteem, according to the proposed model, increases the risk of anxiety and depression disorders, as can be seen below:

- The indirect effect (social media addiction → self-esteem → anxiety disorders) is significant (β = 0.259, *p* = 0.006, R^2^ = 0.182);
- The indirect effect (social media addiction → self-esteem → depressive disorder) is significant (β = 0.291, *p* = 0.010, R^2^ = 0.237).

In order to assess the significance of the mediation model, the bootstrap resampling technique was applied with 5000 replications, considering as significant the relationships whose 95% confidence interval does not contain the value 0 (Liu et al., 2023; Kim et al., 2024). In the case of indirect effects, through bootstrap, it was confirmed that self-esteem remains as a mediator of the relationships: social media addiction—self-esteem—anxiety (β = 0.061, 95% CI (0.018–0.105), *p* < 0.001)/depression (β = 0.064, 95% CI (0.015–0.113), *p* = 0.01), given that the bootstrap confidence intervals do not include the value 0. The same situation is repeated in the case of the total effect between social media addiction—anxiety (β = 0.104, 95% CI (0.057–0.151), *p* < 0.001)/depression (β = 0.111, 95% CI (0.056–0.166), *p* < 0.001), the relationships remaining significant even after applying the bootstrap technique.

The results presented above confirm both hypothesis H1 and hypothesis H3 (see also Figure 1).

In relation to the moderators age and professional status, the results confirmed that the interaction of social media addiction × age is significant (β = 1.101, *p* < 0.001), meaning that age influences the effect of social media addiction on self-esteem. In other words, older people are less affected by the negative impact of social media use on self-esteem. On the other hand, the negative effect is higher for young people, meaning that social media addiction decreases self-esteem more.

The interaction of social media addiction × professional status (β = −0.501, *p* = 0.029) is also significant, suggesting that professional status influences the effect of social media addiction on self-esteem. More specifically, people with a higher professional status (coded in the study database as the following: retired/pensioners, housewives and unemployed) are more vulnerable to decreased self-esteem caused by social media use. In contrast, for those with a lower professional status (coded in the study database as the following: students, employees and freelancers), the negative impact of social media on self-esteem is lower.

Considering the analysis of the relationship (social media addiction–self-esteem) on various levels of moderators, Simple Slope Analysis was used in lavaan, and the level moderation test and the following results were obtained:

(a) The moderator age: The largest negative effect of social media addiction on self-esteem is found in young people (−1 SD) (b = −0.282, 95% CI (−0.446, −0.117), *p* = 0.001), while, for the middle-aged ones (0 SD), the effect remains significant but is slightly smaller (b = −0.273, 95% CI (−0.435, −0.111), *p* = 0.001). For older people (+1 SD), the negative effect is the weakest (b = −0.264, 95% CI (−0.423, −0.105), *p* = 0.001), which suggests that they are more resistant to the negative

(b) The moderator professional status: For students (b = −0.220, 95% CI (−0.414, −0.027), *p* = 0.026), the negative effect of social media addiction on self-esteem is significant but lower than the following two categories of people: for employees and freelancers (b = −0.273, 95% CI (−0.435, −0.111), *p* = 0.001), the negative effect of social media on self-esteem is moderate, while, for unemployed, housewives and retired/pensioners, the negative effect of the relationship is the strongest (b = −0.325, 95% CI (−0.464, −0.186), *p* < 0.001).

These results, in turn, confirm hypotheses H2 and H4 (age and professional status moderate the effect of social media addiction on self-esteem and, implicitly, the mediated effect on anxiety/depression).

## 4. Discussion

Social media addiction includes a combination of cognitive and behavioral symptoms which, together, lead to a series of negative consequences (Davis, 2001) and which can affect mental health. Keeping this direction, the aim of the current study was to analyze the relationship between social media addiction and the manifestation of anxiety and depression symptoms, on a batch of 217 adult Romanian citizens, taking into account the mediator self-esteem, as well as evaluating the entire relationship, subsequently, under the influence of the moderators’ age and professional status.

The descriptive statistical analysis (see Table 1) showed that there were significant correlations with moderate values between the main variables of this study, supporting the research hypotheses. An intense use of social networks was associated with higher levels of anxiety (r = 0.281, *p* < 0.001) and depression (r = 0.327, *p* < 0.001), while the correlations between self-esteem and social network addiction (r = 0.302, *p* < 0.001), anxiety (r = −0.391, *p* < 0.001) and depression (r = −0.443, *p* < 0.001) suggest a possible mediation effect (for the relationships: social network addiction—anxiety/depression disorders). As for the moderators, age and professional status, also from the descriptive statistics (Table 1), we can observe weak or insignificant relationships between them and the predictor (social media addiction–age: r = −0.262, *p* < 0.001);, social media addiction–professional status: insignificant relationship), thus, the two moderators are methodologically validated.

Hypotheses H1 and H3 of the analyzed model, which aimed to test the relationships between social media addiction and the manifestation of symptoms of anxiety disorders (H1)/depression (H3), relationships mediated by self-esteem, were confirmed. The results of the model proposed in this research suggest that people who spend a lot of time on social media tend to have lower self-esteem (Valkenburg et al., 2021; Bergagna & Tartaglia, 2018), which makes them more vulnerable to anxiety (Fernandes et al., 2022; W. Li et al., 2023). This phenomenon can be explained by excessive social comparison (Jiang & Ngien, 2020) as well as by the external validation sought on these platforms (Ballara, 2023; Beyari, 2023; Goodman et al., 2021). At the same time, constant exposure to the seemingly ideal lives of others on social media can lead to feelings of inadequacy and personal devaluation associated with decreased self-esteem (Affizie, 2024). This whole context increases the risk of manifesting depressive symptoms (Lee et al., 2020; Zsila & Reyes, 2023).

Also, hypotheses H2 and H4 were validated, following the statistical analysis, confirming that age and professional status moderate the mediating effect of self-esteem between social media addiction and the manifestation of symptoms of anxiety disorders (H2)/depression (H4). The results of the statistical analysis highlight the fact that young people, namely, those most exposed to digital influences (Eurostat, 2024), are more vulnerable to the negative effects of social media on self-esteem, compared to middle-aged or older people. This vulnerability can be explained by the fact that young people are in a stage of identity development, in which social validation plays an important role (Wilska et al., 2023; Pérez-Torres, 2024). At the same time, decreased self-esteem increases the predisposition to the development of depressive disorders, as already mentioned previously. In contrast, the lower vulnerability observed among middle-aged and older adults can be explained by several psychological and behavioral protective factors, as detailed below. Middle-aged and older people generally have a more stable self-image and a consolidated value system, basing their self-esteem on accumulated experiences, personal achievements and relationships built over time, not only on reactions received from the online environment (Ogihara & Kusumi, 2020; Butkovic et al., 2020). In addition, these people (middle-aged and older) have developed more effective mechanisms for managing stress and social pressures (Aldwin et al., 2021), compared to young people, as they are less susceptible to the negative impact of social comparison (Callan et al., 2015). Another distinction between age categories is represented by the purpose of using social networks, implicitly and subsequently generating an increased impact on self-esteem and anxiety or depression disorders. While adolescents and young adults use social networks frequently for socialization, social comparison and personal validation (Barry et al., 2022; Çınar, 2023), older people are inclined to use them for information, maintaining contacts with family or friends and less to compare themselves with other people (Ractham et al., 2022; Khoo & Yang, 2020).

If social media addiction negatively affects self-esteem in all age groups, but the impact is stronger in the case of young people and lower in the case of older people, with regard to the moderator professional status, although the impact also applies to all professional categories, then the hierarchy is reversed this time. This pattern, opposite to the one observed for age, may appear counterintuitive at first glance and therefore requires a closer look at possible underlying mechanisms. Therefore, analyzed by impact levels, the moderator professional status within the analyzed sample acts as follows: the impact is stronger in the case of the following people: unemployed, housewives and retired/pensioners, moderate in the case of employed and self-employed people, and low in the case of students. These results highlight that people without an active job may face a lack of professional validation, intense social comparisons and exposure to content potentially harmful to self-esteem, the increased frequency of mental illnesses (Suphan et al., 2012), given the willingness to spend more time online (Feuls et al., 2014, 2016). On the other hand, pensioners or retired people may experience a decrease in social status (Grünwald et al., 2022; Kuball & Jahn, 2024), and images of success promoted in the online environment may affect self-esteem, which is already in decline at this stage of life (De Prada et al., 2024). In their turn, employed and self-employed people are less willing to spend time online, outside of strictly professional purposes, and self-esteem, in this case, is generally based on professional achievements, skills and financial income (Bleidorn et al., 2023; F. Jan et al., 2015), thus diminishing social comparison in the online environment. Concerning young people, students, who are at this stage of life, at the peak of their educational and professional potential, in full academic ascent (Mancini et al., 2015), but, without the pressure to reach certain social or financial standards, they do not feel the effect of the intensive use of social networks on self-esteem as strongly as the other aforementioned categories. Professional status acts as a mitigating moderator in the case of students.

Considering the elements presented above, we can infer that the contextual factors, such as age and professional status, may function as protective factors against anxiety and depression disorders caused by the intensive use of social networks. In other words, age and professional status, depending on the steps presented above, mitigate the negative effects of social media addiction on mental health.

The results of the current study are consistent with the specialized literature. For example, in the meta-analysis (Seabrook et al., 2016) conducted on the basis of 70 scientific papers, it was found that negative interactions and social comparisons in the online environment, with a predilection for social networks, are directly related to higher levels of depression and anxiety. Other studies have highlighted the fact that, among adolescents and young people, the use of social networks can result in decreased self-esteem (Bell, 2019), self-harm (Biernesser et al., 2020), depression and anxiety (Keles et al., 2020). Similar results can be found in the literature on the adult, middle-aged, professionally active population: the manifestation of anxiety disorders and depression (Lin et al., 2016; Lambert et al., 2022).

Although these findings are consistent with results from international studies, it is important to acknowledge the national context. In Romania, cultural attitudes toward social media, mental health, and social comparison may shape how individuals, especially across different age and professional groups, internalize online interactions.

These patterns may also be interpreted through the lens of self-determination theory (Ryan & Deci, 2020), which suggests that variations in autonomy and competence—linked to age and professional status—can shape how individuals experience the psychological effects of social media. This framework helps contextualize the moderation effects observed in the current study.

Considering the digital influences on mental health, the results of this study emphasize, first of all, the importance of developing strategies for the conscious use of social networks and forming an authentic, coherent and balanced self-image based on internal factors.

## 5. Limitations and Future Research Directions

The current study, descriptive, exploratory, differential and correlational, makes an important contribution to the specialized literature, first of all through the features of the chosen batch (Romanian citizens, adults), considering the existence of a very small number of studies with these characteristics. Secondly, the particularities of the current research highlighted by the combination of the analyzed variables (social media addiction, self-esteem, depression and anxiety disorders, age and professional status), also represent, at least for Romania, a new approach on the topic of social media addiction and mental health.

However, the current study also has a number of limitations. The first limitation is related to the study design, which is cross-sectional rather than longitudinal, so it was not possible to identify causal inferences (Lim et al., 2021). Therefore, it is desirable for future studies to adopt a longitudinal design.

Another limitation of this study concerns the sample size (N = 217) used for the SEM analysis. Although the sample exceeds the commonly cited minimum threshold of 200 for SEM (Kline, 2015; Hair et al., 2010), more complex models that include multiple latent constructs, mediation, and moderation paths typically benefit from larger samples to ensure the stability and generalizability of parameter estimates. While our sample size was adequate for detecting average effect sizes and was determined a priori using the G*Power program, future studies should aim to replicate and extend the current findings using larger and more diverse populations.

In addition to the sample size, the demographic composition also presents limitations. The sample included more women, urban residents, and individuals with higher education. This should be taken into account, and future research would benefit from including participants with more diverse backgrounds.

It is also necessary to add that this study relied on self-administered questionnaires, which are subject to bias, leading to the possibility that participants did not provide accurate or truthful answers, a situation that can generate measurement distortions (Smith, 2024). Future studies could use physiological measurement instruments instead of questionnaires for greater certainty.

We also consider that another important limitation is that, in the current study, reference was made to social media, in general, and not to social media specifically (e.g., Instagram, Facebook, TikTok, etc.). Therefore, future studies will need to explore the specific influence of social media on mental health while also taking into account mediators or/and moderators not included in this study, for example, social support, the level of education, etc.

In conclusion, given the increasing prevalence of mental health problems, we consider it essential to include the topic of social media addiction and its impact on mental health in the agendas of researchers in Romania. This need is supported by the results of the current study, which showed, as previously mentioned, that the proposed model explains 18.2% of the variation in anxiety disorders and 23.7% of the variation in depression disorders, based on the relationship between social media addiction and the manifestation of anxiety and depression symptoms, a relationship explained by self-esteem, under the influence of age and professional status.

## Figures and Tables

**Table 1:** Descriptive statistics and correlations between the investigated variables.

	Mean	SD	1	2	3	4	5	6	7
Anxiety	5.11	4.59	—						
Depression	3.84	4.25	0.58 ***	—					
Self-esteem	32.22	6.86	−0.391 ***	−0.443 ***	—				
Social media addiction	56.85	19.35	0.281 ***	0.327 ***	0.302 ***	—			
Professional status	2.03	0.77	−0.095	−0.104	−0.126	−0.033	—		
Age	36.15	11.92	−0.081	−0.208 **	0.353 ***	−0.262 ***	0.387 ***	—	
Gender			0.189 **	0.039	0.006	−0.017	0.292 ***	0.37 ***	—

Note: ** *p* < 0.01, *** *p* < 0.001; 1 = anxiety, 2 = depression, 3 = self-esteem, 4 = social media addiction, 5 = professional status, 6 = age, 7 = gender; and SD = standard deviation.

## Data Availability

The original contributions presented in this study are included in this article, and further inquiries can be directed to the corresponding author.

## Abbreviations

The following abbreviations are used in this manuscript:

b	Unstandardized regression coefficient
β	Beta (the standardized path coefficient between latent variables)
CFI	Comparative Fit Index
CI	Confidence interval
CMINF/DF	Chi-square/Degrees of Freedom ratio
DASS-21R	Depression, Anxiety, and Stress Scale—21 Revised
FOMO	Fear of Missing Out
H	Hypothesis
*p*	*p*-value (statistical significance)
r	Pearson correlation coefficient
R^2^	Coefficient of Determination
RMSEA	Root Mean Square Error of Approximation
SD	Standard deviation
SEM	Structural Equation Modeling
SRMR	Standardized Root Mean Square Residual
TLI	Tucker–Lewis Index
